# Vitamin D deficiency is common in psychogeriatric patients, independent of diagnosis

**DOI:** 10.1186/1471-244X-14-134

**Published:** 2014-05-08

**Authors:** Ole Grønli, Jan Magnus Kvamme, Rolf Jorde, Rolf Wynn

**Affiliations:** 1 Department of Clinical Medicine, Faculty of Health Sciences, University of Tromsø, Tromsø, Norway; 2 University Hospital of North Norway, Tromsø, Norway; 3 Department of Geriatric Psychiatry, UNN-Åsgård, Tromsø N-9291, Norway

## Abstract

**Background:**

Previous studies have found an association between psychiatric disorders and vitamin D deficiency, but most studies have focused on depression. This study aimed to establish the prevalence of vitamin D deficiency in elderly patients with a wider range of psychiatric diagnoses.

**Method:**

The study included elderly patients (>64 years) referred to a psychiatric hospital in Northern Norway and a control group from a population survey in the same area. An assessment of psychiatric and cognitive symptoms and diagnoses was conducted using the Montgomery and Aasberg Depression Rating Scale, the Cornell Scale for Depression in Dementia, the Mini Mental State Examination, the Clockdrawing Test, and the Mini International Neuropsychiatric Interview (MINI+), as well as clinical interviews and a review of medical records. The patients’ mean level of 25-hydroxyvitamin D (25(OH)D) and the prevalence of vitamin D deficiency were compared with those of a control group, and a comparison of vitamin D deficiency across different diagnostic groups was also made. Vitamin D deficiency was defined as 25(OH)D <50 nmol/L (<20 ng/ml).

**Results:**

The mean levels of 25(OH)D in the patient group (n = 95) and the control group (n = 104) were 40.5 nmol/L and 65.9 nmol/L (p < 0.001), respectively. A high prevalence of vitamin D deficiency was found in the patient group compared with the control group (71.6% and 20.0%, respectively; p < 0.001). After adjusting for age, gender, season, body mass index, and smoking, vitamin D deficiency was still associated with patient status (OR: 12.95, CI (95%): 6.03-27.83, p < 0.001). No significant differences in the prevalence of vitamin D deficiency were found between patients with different categories of psychiatric diagnoses, such as depression, bipolar disorders, psychosis, and dementia.

**Conclusion:**

Vitamin D deficiency is very common among psychogeriatric patients, independent of diagnostic category. Even though the role of vitamin D in psychiatric disorders is still not clear, we suggest screening for vitamin D deficiency in this patient group due to the importance of vitamin D for overall health.

## Background

There has been an increase in interest in vitamin D deficiency over the last 10 years. Several studies have revealed a high prevalence of vitamin D deficiency even in areas of the world that receive ample sunlight. The level of 25-hydroxyvitamin D (25(OH)D) is used to evaluate subjects’ vitamin D status. In Australia, India, and Saudi Arabia, 30-50% of children and adults have 25(OH)D levels below 50 nmol/L [1-3]. Similarly, in Norway, more than 40% of the population have been reported to have serum 25(OH)D levels below 50 nmol/L [4]. Only a few types of food naturally contain vitamin D (e.g., salmon, mackerel, and cod liver oil); therefore, the major source of vitamin D is sunlight [5]. UVB radiation in the 290–315 nm wavelength converts 7-dehydrocholesterol in the skin to previtamin D3, which, in turn, is converted into vitamin D3. Vitamin D3 is then hydroxylated in the liver to 25(OH)D and further hydroxylated in the kidneys to its active form, 1,25 dihydroxyvitamin D (1,25(OH)_2_D) [6]. Latitude and season affect the quantity and quality (wavelength) of solar radiation and thus influence the ability of sunlight to synthesize vitamin D3 in the skin [7]. For example, in Edmonton, Canada (52°N), skin exposed to sunlight from October through March produced no previtamin D3 [7]. Previous studies showed that elderly individuals produce only 25% of the cutaneous vitamin D produced by young adults [8].

There has been some debate concerning the optimal range of 25(OH)D. Serum levels of 25(OH) D below 50 nmol/L are associated with an increase in serum parathyroid hormone (PTH) levels [9] and a decrease in physical performance in older individuals [10]. It has been suggested that serum 25(OH)D levels above 50 nmol/L are sufficient to sustain bone density and calcium absorption and to prevent osteomalacia [11]. This cut-off level is now widely used in studies of vitamin D deficiency.

Several cross-sectional studies have found an association between vitamin D deficiency and depression or depressive symptoms [12-16]. Despite some negative reports [17], Anglin et al. [18] concluded in a recent meta-analysis that low vitamin D concentration is associated with depression, but further randomized controlled trials (RCTs) of vitamin D are needed to determine whether this association is causal. There have been a few RCTs studying the effects of vitamin D on depressive symptoms, but the findings are inconsistent [19-21]. A recently published RCT did investigate the effects of vitamin D supplementation as adjuvant treatment in patients with major depressive disorder. The results showed a significant improvement in patients being treated with an antidepressant and vitamin D as compared with patients receiving an antidepressant and placebo [22]. There are also studies reporting an association between vitamin D deficiency and cognitive impairment [23-25] and even psychosis has been associated with vitamin D deficiency [26-28].

Psychogeriatric patients may be at risk for vitamin D deficiency due to their age, less exposure to sunlight, and dietary factors. However, there is little knowledge regarding the actual vitamin D status in this group of patients. Therefore, one aim of this study was to compare the level of 25(OH)D in a sample of psychogeriatric patients with the level in the general elderly population from the same area. In addition, the study aimed to examine whether patients with depression differ from patients with other psychiatric diagnoses with regard to vitamin D status.

## Methods

### Study population

The study took place from March 2010 to December 2011. Patients aged 65 years or older who were referred to a psychiatric hospital located in Tromsø (69°N) in the northern part of Norway were eligible for inclusion. The hospital has a population base of 255,000 subjects. Patients who were not able to communicate due to their medical condition (i.e., severe dementia) were excluded. With regard to the controls, subjects older than 64 years who had participated in a follow-up study after the 6th Tromsø study and had normal glucose tolerance were randomly selected and stratified for gender, age, BMI, and blood sampling season.

### Measures

#### Assessment of psychiatric symptoms

The following instruments were used to diagnose the participant patients: the Mini International Neuropsychiatric Interview (MINI+) [29], the Montgomery-Asberg Depression Rating Scale (MADRS) [30], the Mini Mental Status Examination (MMSE) [31], and the Clockdrawing Test [32]. In addition to the structured diagnostic psychiatric interview (MINI+), clinical interviews and medical records were also used to diagnose the participants. Patients with dementia (n = 19) who could not participate in a MADRS interview were assessed with the Cornell Scale for Depression in Dementia [33]. Diagnoses were made according to ICD-10 criteria (WHO, 1992). The control group was not assessed for the presence of psychiatric disorders.

#### Laboratory analyses

Blood samples for 25(OH)D were drawn in the morning before 10 AM during the first 3 days of the hospital stay and were stored at -70°C. Analyses of 25(OH) D were performed using a competitive radioimmunoassay (RIA) (Dia Sorin, Stillwater, MN) [34]. Vitamin D deficiency was defined as 25(OH) D levels <50 nmol/L. In addition, analyses of calcium, albumin, PTH, creatinine, erythrocyte sedimentation rate (ESR), C-reactive protein (CRP), glucose, free thyroxine (FT4), and TSH were performed the same day.

#### Statistical analyses

The SPSS 20 software package (SPSS Inc., Chicago, Illinois, USA) was used for statistical analyses. The Kolmogorov-Smirnov test was used to confirm assumptions of normal distributions. Baseline differences were analyzed using chi-square tests (dichotomous data), Mann–Whitney U-test and independent samples t-tests (continuous data). Logistic regression models were used to assess the association between vitamin D deficiency and the sample variables (patient vs. control), controlling for potential confounders. The results were expressed as odds ratios (ORs) with 95% confidence intervals (CIs). The association of vitamin D deficiency status with psychiatric diagnoses was examined using the Fisher’s exact test. A correlation between PTH and 25(OH)D was calculated using Pearson’s correlation method. A *p-*value < 0.05 was accepted as statistically significant. The data are presented as the mean (SD) unless otherwise specified.

### Ethics and consent

Possible candidates for inclusion in the study were given oral and written information about the study. The next of kin was given similar information if a patient was unable to provide informed consent alone due to his/her medical condition. Written consent (from the patient or the next of kin) was provided prior to inclusion in the study. Existing guidelines were used to assess competency to give consent [35]. The Regional Medical Ethics Committee for North Norway (REK North) approved the study.

## Results

### Clinical characteristics

A total of 95 patients (90 inpatients and 5 outpatients) and 104 controls were included in the study, all of whom were ethnic Norwegians. Basic characteristics of the patient and control groups are shown in Table 1. The patients suffered from a range of disorders that are typical in a psychogeriatric population (Table 2). We found that 40 patients had a depressive disorder, either as a first-time depressive episode, recurrent depression or as part of a bipolar disorder. We also identified 18 patients with depression secondary to other diagnoses (e.g., dementia or organic mood disorder). In 37 of the patients no depressive disorders were identified. In this sub-group, 18 patients had dementia (14 with Alzheimer’s disease), 11 patients had a psychotic disorder, and 8 patients had other non-depressive disorders.

**Table 1:** Clinical characteristics of the patient and control groups

	**Patients**	**Controls**	***p*-value**
Gender % (female/male)	63.2/36.8	62.9/37.1	*p* = 0.965*
Age, mean (SD)	77.2 (6.5)	74.6 (1.3)	*p* = 0.002**
Smoking (%)	29.3	17.1	*p* = 0.042*
BMI (kg/m^2^), mean (SD)	25.3 (5.4)	25.9 (4.1)	*p* = 0.330***
Blood sampling season (winter, spring, summer, autumn) (%)	18/28/26/27	25/28/28/20	*p* = 0.515*

*Notes:* *χ^*2*^ *=* Chi-square test, **Mann–Whitney U-Test, ***Student’s t-test.

**Table 2:** Diagnoses of the 95 patients participating in the study

**Diagnoses**	**N**
Dementia	
Alzheimer’s	20
Vascular	7
Other dementia	4
Other organic mental disorders	4
	
Depressive episode	5
Recurrent depression	26
Bipolar affective disorders	
Depression	8
Manic	2
Psychotic disorders	12
Anxiety disorders	4
Other diagnoses	3

### Serum 25(OH)D levels

The mean (SD) serum 25(OH)D levels were 40.5 (19.0) nmol/L, which was significantly lower than the control group’s of 65.9 (19.5) nmol/L (t(198) = -9.19, *p* < 0.001). In the patient group, 68 patients (71.6%) had vitamin D deficiency (serum 25(OH)D <50 nmol/L), as compared with only 21 individuals (20.0%) in the control group (χ^2^ = 53.7, p < 0.001). Severe vitamin D deficiency (25(OH)D <25 nmol/L) was observed in 18 patients (19.4%) and 2 individuals (1.9%) in the control group (p < 0.001).

There was no significant gender difference between the mean 25(OH)D levels neither in the patient group (women: 40.7 nmol/L, men: 40.3 nmol/L) or in the control group (women: 63.3 nmol/L, men: 69.4 nmol/L). In a multivariate model that included age, gender, BMI, smoking, and blood sampling season, only patient status and blood sampling season were predictive of vitamin D deficiency (Table 3).

**Table 3:** Adjusted odds ratio (95% CI) for the association between vitamin D deficiency and patient/control status

	**OR (95% CI)**	***p***
Patient/control	12.95 (6.03-27.83)	*p* <0.001
Sex	1.55 (0.76-3.16)	*p* = 0.231
Age	0.99 (0.92-1.07)	*p* = 0.876
Winter*		*p* = 0.067
Spring	0.61 (0.23-1.62)	*p* = 0.319
Summer	0.27 (0.10-0.75)	*p* = 0.012
Autumn	0.36 (0.13-1.04)	*p* = 0.060
BMI	0.94 (0.87-1.83)	*p* = 0.085
Smoking	0.79 (0.34-1.83)	*p* = 0.581

Notes: *season for blood sampling.

### Serum 25(OH)D levels and psychiatric diagnoses

**Figure 1:**
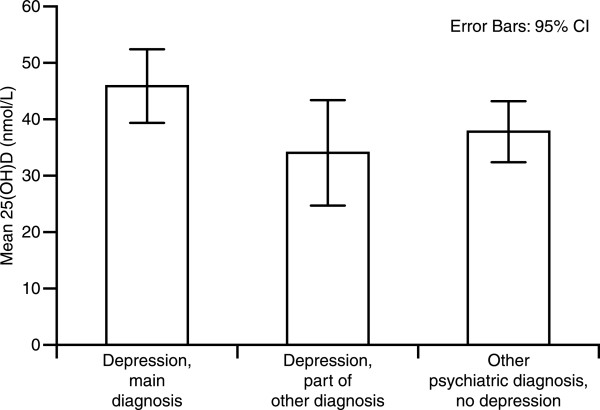
Serum 25(OH)D levels (nmol/L) in subjects with depression, comorbid depression, and other psychiatric diagnoses.

In the patient group, we did not find any significant difference in 25(OH)D levels between patients with depression as the main diagnosis, patients with depression as part of other diagnoses, and patients with another psychiatric diagnosis (Figure 1). Furthermore, when grouping the patients into five diagnostic categories (unipolar depression, bipolar depression, psychosis, dementia, and other diagnoses), there were still no significant differences in serum 25(OH)D levels between the groups (Table 4). There was a small, but significant correlation between MADRS score and serum 25(OH)D levels (r_s_ = .26, p = 0.028).

**Table 4:** Serum 25(OH) D levels and prevalence of vitamin D deficiency in relation to diagnoses

**Diagnoses**	**N**	**25 (OH) level (nmol/L)**^**1**^	**Prevalence (%) of vitamin D deficiency**^**2**^
Depression	31	45.9 (20.5)	71.0
Bipolar disorder	10	43.8 (18.0)	50.0
Psychosis	12	33.9 (16.0)	83.3
Dementia	31	38.6 (18.6)	74.2
Other diagnoses	11	34.8 (17.6)	72.7

^1^A one-way ANOVA test revealed no significant difference (F(4,90) = 1.41, *p* = 0.236) in 25(OH)D levels between the different diagnostic groups.

^2^The Fisher’s exact test indicated no significant association (χ^2^ = 3.1, *p* = 0.522) between vitamin D deficiency and diagnosis.

**Figure 2:**
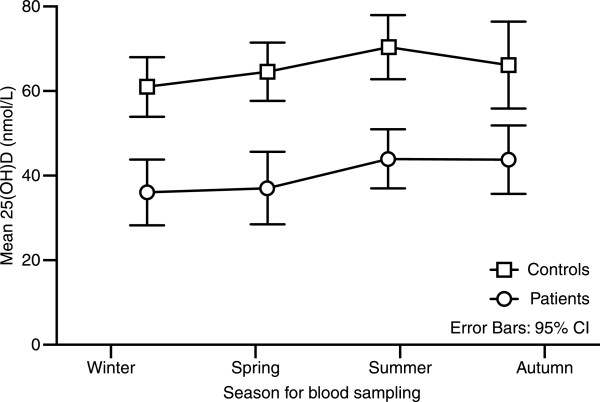
Serum 25(OH)D levels in relation to season.

In the patient group, we had data on vitamin D sources used, namely, cod fish oil (a common supplement in Norway), vitamin D tablets (multivitamin), or vitamin D-fortified milk. We found that 39 patients (41%) took some type of vitamin D supplement, 44 patients (46%) did not take any supplement, and vitamin D source data were missing for 12 patients. There was no significant difference in 25(OH)D levels between the patient group that reported an intake of extra vitamin D and the group with no intake of extra vitamin D (mean 25(OH)D levels were 42.2 nmol/L and 36.2 nmol/L, respectively). Furthermore, there was no significant association between vitamin D deficiency and intake of a vitamin D supplement. We also had data on the average time spent outdoors over the 3 months before study inclusion. There was no significant difference in 25(OH)D levels between patients who spent less than 30 min outdoors every day (n = 61, mean serum 25(OH)D = 39.5 nmol/L (17.2)) and patients who spent more than 60 min outside every day (n = 17, serum 25(OH)D = 34.0 nmol/L (14.0)). As expected, there were seasonal variations in vitamin D status, with higher levels present in summer and autumn. This result was a trend in both the patient and control groups (Figure 2), but the differences in serum 25(OH)D level between different seasons were not statistically significant.

A total of 26% of the patients had elevated PTH levels (>7.5 pmol/L), and there was a negative correlation between serum 25(OH)D and PTH levels (r = -0.34, *p* < 0.001). We did not have data on PTH in the control group. Neither the correlation between serum 25(OH)D levels and MMSE (r_s_ = -.11, p = 0.297) or with Clockdrawing Test scores in the patient group (r_s_ = .07, p = 0.508) were significant.

## Discussion

In this study, we found a high prevalence (71.6%) of vitamin D deficiency in elderly psychiatric patients compared with a control group (19.2%). We could not find any difference in vitamin D deficiency between patients in different diagnostic groups. Although this study was conducted in the northern part of Norway, vitamin D deficiency is a common problem in other parts of the world as well. Van der Wielen et al. found that among the elderly, vitamin D deficiency was more common in southern Europe than in Scandinavia [36]; therefore, our findings seem to be relevant globaly.

Our results are in line with those of other studies. In a group of psychogeriatric inpatients, Lapid et al. found no association between 25(OH)D level and psychiatric diagnosis [37], and the same findings were reported in a non-elderly group [38]. A Swedish study of adult psychiatric outpatients reported low levels of 25(OH)D (median 45 nmol/L), with slightly lower levels in patients with schizophrenia and autism compared with other diagnoses [39]. However, we are not aware of any studies that have examined the difference in levels of 25(OH)D between a control group and psychogeriatric patients with a wide range of psychiatric disorders.

There are several possible explanations for our findings. On the one hand, it is possible that older individuals with psychiatric disorders have a diet and outdoor activity pattern that may result in vitamin D deficiency, which could also be the case for different diagnostic groups, not only depressed patients. The hospital associated with this study is located at high latitude in the northern part of Norway, where there is no sun-induced production of vitamin D in the skin for more than 6 months of the year [7]. This patient group could thus be even more vulnerable to vitamin D deficiency due to a diet low in vitamin D. On the other hand, one cannot rule out the possibility that vitamin D deficiency can contribute to the development of several psychiatric disorders. It is well known that a diversity of psychiatric symptoms can occur in connection with various medical conditions. Some patients with thyrotoxicosis develop depression, others develop anxiety or psychosis, and some do not develop psychiatric disorders at all [40]. The same pattern is found in patients with hyperparathyroidism [41] and in patients with low levels of vitamin B12 [42]. These results suggests that there could be some unknown individual factors that make some people more vulnerable than others to the development of various psychiatric symptoms due to alterations in these hormones and micronutrients. Although we could not find a significant difference in serum 25(OH)D levels between different diagnostic groups, we can not rule out the possibility of a type II error, due to modest number of subjects in some of our diagnostic sub-groups.

Several human and animal studies have suggested possible biological explanations for the importance of vitamin D in psychiatric disorders. Vitamin D receptors and the enzyme required for its activation, 1α hydroxylase, have been identified in the human brain [43]. Active vitamin D (1,25(OH)_2_D) is shown to alter cholinergic, dopaminergic, and noradrenergic neurotransmitter systems in animal models [44]. Abnormalities of these neurotransmitters have been linked to various neuropsychiatric disorders [45]. Groves et al. studied the impact of adult vitamin D deficiency in two strains of mice [46], and a small reduction in an enzyme involved in GABA synthesis (GAD65/67) was reported. Altered GABAergic neurotransmission has been linked to several psychiatric conditions, including schizophrenia [47], anxiety [48], and depression [49]. In the most neophobic strain (BALB/c mice), Groves et al. also found alterations in glutamate and glutamine levels in brain tissue, which may indicate a disruption in glutaminergic neurotransmission. NMDA receptor (glutamate receptor) dysfunction has been linked to several psychiatric conditions, including depression and schizophrenia [50,51].

As expected, there was a significant negative correlation between serum 25(OH)D and PTH levels [9,52]. High levels of PTH have been associated with impaired performance in cognitive tests [53] and with depression [52]. Primary hyperparathyroidism with elevated PTH and calcium can present with a variety of psychiatric symptoms [41], and we previously reported that this may be due to elevated PTH and not elevated calcium [54]. It is therefore possible that an effect of vitamin D on psychiatric symptoms could be indirectly caused by elevated PTH levels.

There was no significant difference in vitamin D levels between patients who reported taking a vitamin D supplement and patients who reported not taking a vitamin D supplement. These data are based on patients’ self-reporting and thus it is difficult to draw any firm conclusions. However, the finding could strengthen the impression that the recommended supplement dose of vitamin D in Norway (400 IE vitamin D_3_) is not enough to maintain an adequate level of vitamin D in elderly individuals, and several authors have recommended 800–1000 IE vitamin D_3_ as a daily supplement for the elderly [6,55,56].

Although the role of vitamin D in psychiatric disorders is not clear, there are also other reasons to be aware of vitamin D deficiency. A meta-analysis of 17 randomised trials demonstrated a 12% risk reduction in fractures in patient groups receiving calcium and vitamin D supplements [57]. Vitamin D deficiency can cause proximal muscle weakness and muscle pain [58]. Vitamin D supplements increase muscle strength and balance and reduce the risk of falling [59,60]. In observational studies, vitamin D deficiency has also been associated with an increased risk of several other conditions, including cancer, diabetes, and multiple sclerosis, as well as a higher overall mortality rate and premature aging [6,61-63]. However, a recent review by Autier et al., found that intervention studies did not show an effect of vitamin D supplementation on non-skeletal health outcome whereas observational studies did. An exception was slightly reduced all-cause mortality [64]. The authors proposed that this difference in outcome might indicate that low 25(OH)D is a marker for ill health [64].

Our current study has some limitations. A reverse causality cannot be ruled out in this type of study design, as we do not know whether vitamin D deficiency is a contributor or a consequence of psychiatric disorders. In the control group, we did not have data to assess medical comorbidity, and therefore, we were not able to control for ill health as a possible confounder. We did not have data on the amount of time spent outdoors, use of vitamin D supplements, or psychiatric symptoms in the control group. It is unlikely, however, that elderly patients with significant mental distress participated in the population survey. Hansen et al. found that non-attendees in a survey had a 2.5-fold higher prevalence of psychiatric disorders compared with attendees [65]. In the patient group, there may also have been some recollection bias when the patients were asked about vitamin D supplementation and time spent outdoors.

## Conclusions

Although our study did not address the issue as to whether the observed differences represent a causal relationship between vitamin D deficiency and psychiatric symptoms, the results are nonetheless important given the significance of vitamin D to overall health. We suggest that increased attention should be given to vitamin D deficiency in elderly patients with psychiatric disorders. Serum 25(OH)D assessment should be included in the set of standard blood tests performed in this patient group and treatment with vitamin D should be initiated in patients with low serum 25(OH)D. There is mounting evidence suggesting that vitamin D could have an impact on a variety of psychiatric conditions; however, more RCTs are needed to address the question of causality.

## Competing interests

The authors declared that they have no competing interests.

## Authors’ contributions

OG and RW designed the study. OG collected the data and conducted the data analyses. RJ, RW and JMK contributed to the interpretation of the data. OG wrote the first draft of the manuscript. RJ, RW and JMK reviewed and revised the manuscript. All authors have read and approved the final version of the manuscript.

## Pre-publication history

The pre-publication history for this paper can be accessed here:

http://www.biomedcentral.com/1471-244X/14/134/prepub
